# The pro-inflammatory cytokine 14-3-3ε is a ligand of CD13 in cartilage

**DOI:** 10.1242/jcs.169573

**Published:** 2015-09-01

**Authors:** Meriam Nefla, Laure Sudre, Guillaume Denat, Sabrina Priam, Gwenaëlle Andre-Leroux, Francis Berenbaum, Claire Jacques

**Affiliations:** 1 UMR_S938, CDR Saint-Antoine - INSERM - University Pierre & Marie Curie Paris VI, Sorbonne Universités, 7 quai St-Bernard, Paris 75252, Cedex 5, France; 2 Inflammation-Immunopathology-Biotherapy Department (DHU i2B), |a184 rue du Faubourg Saint-Antoine, Paris 75012, France; 3 INRA, Unité MaIAGE, Mathématiques et Informatique Appliquées du Génome à l'Environnement, UR1404, Jouy-en-Josas F78352, France; 4 Department of Rheumatology, Assistance Publique – Hôpitaux de Paris, Saint-Antoine Hospital, 184 rue du Faubourg Saint-Antoine, Paris 75012, France

**Keywords:** 14-3-3ε, CD13, Aminopeptidase N, Chondrocyte, Osteoarthritis

## Abstract

Osteoarthritis is a whole-joint disease characterized by the progressive destruction of articular cartilage involving abnormal communication between subchondral bone and cartilage. Our team previously identified 14-3-3ε protein as a subchondral bone soluble mediator altering cartilage homeostasis. The aim of this study was to investigate the involvement of CD13 (also known as aminopeptidase N, APN) in the chondrocyte response to 14-3-3ε. After identifying CD13 in chondrocytes, we knocked down CD13 with small interfering RNA (siRNA) and blocking antibodies in articular chondrocytes. 14-3-3ε-induced MMP-3 and MMP-13 was significantly reduced with CD13 knockdown, which suggests that it has a crucial role in 14-3-3ε signal transduction. Aminopeptidase N activity was identified in chondrocytes, but the activity was unchanged after stimulation with 14-3-3ε. Direct interaction between CD13 and 14-3-3ε was then demonstrated by surface plasmon resonance. Using labeled 14-3-3ε, we also found that 14-3-3ε binds to the surface of chondrocytes in a manner that is dependent on CD13. Taken together, these results suggest that 14-3-3ε might directly bind to CD13, which transmits its signal in chondrocytes to induce a catabolic phenotype similar to that observed in osteoarthritis. The 14-3-3ε–CD13 interaction could be a new therapeutic target in osteoarthritis.

## Abstract (precis)

**Summary:** 14-3-3 might directly bind to CD13, which transmits a signal in chondrocytes to induce a catabolic phenotype similar to that observed in osteoarthritis.

## INTRODUCTION

Osteoarthritis is a chronic joint disease characterized by a progressive degradation of articular cartilage, synovial inflammation and remodeling of subchondral bone in response to a variety of stimuli, such as proinflammatory cytokines and mechanical loading (Bijlsma et al., 2011; Martel-Pelletier and Pelletier, 2010). The catabolic phenotype observed in osteoarthritis is caused by an imbalance in synthesis and degradation of matrix components, mainly due to increased production of matrix-degradative enzymes, such as matrix metalloproteinases (MMPs) and aggrecanases (Findlay and Atkin, 2014; Felson and Neogi, 2004). Among the MMPs, MMP-3 and MMP-13 are known to play crucial role in osteoarthritis cartilage destruction (Kim et al., 2014).

There is now general agreement that osteoarthritis involves all structures in the affected joint, which leads to articular cartilage deterioration. Several studies have suggested that subchondral bone and articular cartilage form a functional unit, which might play a role in joint maintenance and degeneration. There is a possibility of direct signaling between the two compartments (Imhof et al., 2000; Pan et al., 2009; Clark and Huber, 1990) and that they interact through increased vascularization and development of micro cracks in the bone matrix (Sharma et al., 2013). With respect to cartilage, the diffusion of mediators has been well demonstrated. For example, insulin-like growth factor is largely transported into cartilage from the circulation (Zhang et al., 2010). Hepatocyte growth factor is found in the deep layers of cartilage; it is produced by osteoarthritis osteoblasts and found in cartilage, even though it is not expressed by chondrocytes. These results suggest the passage of this molecule from bone to cartilage (Guevremont et al., 2003).

Recently, our group identified 14-3-3ε as a new soluble mediator involved in deleterious biochemical interactions between bone and cartilage (Priam et al., 2013). Using iTRAQ analysis of conditioned medium from compressed or uncompressed osteoblasts or osteocytes, we found that 14-3-3ε is upregulated in medium from compressed cells. In addition, 14-3-3ε was released by human osteoarthritis subchondral bone and had a potent effect on the pro-catabolic phenotype of human osteoarthritis chondrocytes by increasing MMP release (Priam et al., 2013).

14-3-3ε belongs to the family of 14-3-3 proteins, acidic proteins of 25 to 33 kDa that are highly conserved in eukaryotes (Aitken, 1995). The proteins were first identified by Moor and Perez in 1968 (Moore et al., 1968) in the brain tissue of mammals. Members of the 14-3-3 family are highly related, and at least seven isoforms have been identified in mammal cells (β, ε, η, γ, τ, σ and ζ) (Muslin et al., 1996). 14-3-3 proteins have a large number of partners and thus participate in many intracellular processes including cell cycle control, mitogenesis, cytoskeletal changes and apoptosis (Pallucca et al., 2014; Aitken, 2011). However, they can also be detected in the extracellular environment. The first secreted forms of 14-3-3 were detected in the cerebrospinal fluid of patients with Creutzfeld–Jacob disease (Boston et al., 1982). 14-3-3η and 14-3-3γ proteins were detected in the synovial fluid of patients with rheumatoid arthritis. The levels of these proteins were strongly correlated with levels of MMP-1 and MMP-3, two biomarkers for rheumatoid arthritis (Kilani et al., 2007). It has been determined that 14-3-3σ is involved in the dialog between keratinocytes and fibroblasts during tissue repair. Indeed, 14-3-3σ is abundantly secreted by differentiated keratinocytes, and can induce the expression of MMP-1, MMP-3 and MMP-8 in dermal fibroblasts (Ghahary et al., 2004). Lung-epithelial-cell-derived 14-3-3α and 14-3-3β also has a potent MMP-1-inducing effect on airway fibroblasts (Asdaghi et al., 2012).

No receptor for 14-3-3ε has been identified in articular cartilage. However, CD13 (also known as aminopeptidase N, APN) has recently been identified as a 14-3-3σ receptor on fibroblasts (Ghaffari et al., 2010). Thus, CD13 could be a potential receptor for 14-3-3ε on the chondrocyte surface. CD13 is a 150-kDa surface glycoprotein, and an ectoenzyme of the superfamily of zinc metalloproteases (EC 3.4.11.2) present in many human tissues. CD13 is widely expressed as a homodimer of high molecular mass (∼280 kDa) on the cell surface of tissues such as intestinal epithelia and the nervous system (Zhang et al., 2013). It preferentially cleaves neutral amino acids, most notably alanine residues, from the N-terminus of peptides (Chen et al., 2012). Polypeptide chains of this ectoenzyme are organized in different domains: a small cytoplasmic N-terminal domain, a transmembrane domain and a large extracellular domain containing the active site (Luan and Xu, 2007). CD13 plays pivotal roles in many physiological processes such as antigen-presenting regulation, tumor angiogenesis and metastasis, differentiation, proliferation, apoptosis, chemotaxis, crystallization cholesterol, phagocytosis, pain sensation, cell–cell adhesion and coronavirus entry. CD13 performs these functions through its role as a receptor involved in cellular signal transduction and/or peptide cleavage through its aminopeptidase N enzymatic activity (Mina-Osorio, 2008; Gabrilovac et al., 2005). However, an involvement of CD13 in osteoarthritis has never been shown, although this molecule has been thoroughly investigated in another articular pathology, rheumatoid arthritis (Shimizu et al., 2002). Recently, Morgan and colleagues have suggested that the soluble form of CD13, released into synovial fluid, acts as a T-cell chemoattractant that induces rheumatoid arthritis synovitis (Morgan et al., 2015).

We lack evidence of 14-3-3ε associated with a chondrocyte surface receptor. In the current study, we report the association of 14-3-3ε and CD13, and highlight a transmembrane signaling mechanism for 14-3-3ε-mediated MMP-3 and MMP-13 expression in chondrocytes. This interaction between 14-3-3ε and CD13, leading to a chondrocyte catabolic phenotype similar to that observed in osteoarthritis, could be a new target for treatment of osteoarthritis and could be investigated in preclinical studies.

## RESULTS

### Expression of CD13 in articular chondrocytes

**Fig. 1.**
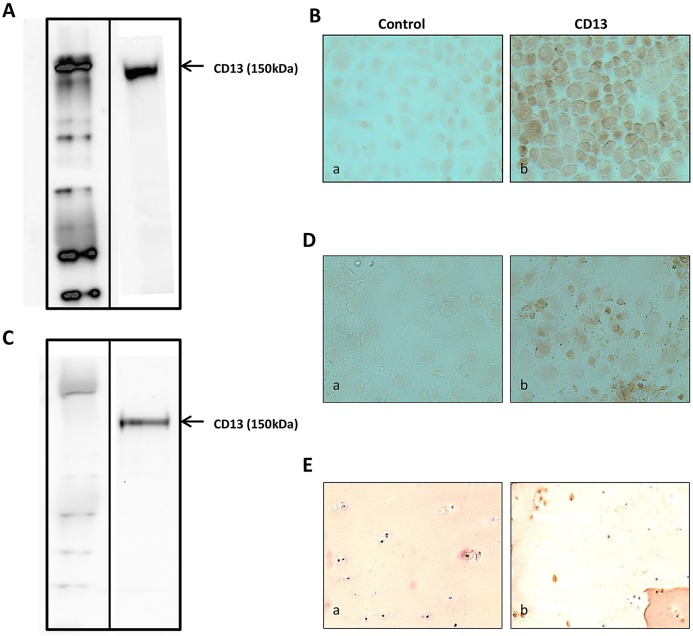
**Expression of CD13 on the surface of murine and human articular chondrocytes.** (A,C) Western blot analysis of CD13 protein expression in mouse (A) and human (C) articular chondrocytes. (B,D) Immunocytochemical detection of CD13 expression in mouse (B) and human (D) chondrocytes with anti-CD13 antibody (AB108310) (a) or rabbit IgG (b) (original magnification ×60). Immunohistochemistry analysis of CD13 expression on the surface of human cartilage (E) with anti-CD13 antibody (AB108310) (a) or rabbit IgG (b) (original magnification ×20).

CD13 expression in articular chondrocytes was first examined in primary cultures of mouse chondrocytes. Using quantitative real-time PCR (qRT-PCR), mRNA expression of CD13 was demonstrated in murine chondrocytes, with the peak of expression at between 24 and 26 cycles (data not shown). CD13 protein expression was found in articular chondrocyte extracts by western blot analysis (Fig. 1A) and immunocytochemistry (Fig. 1B). CD13 mRNA and protein expression was verified in human osteoarthritis articular chondrocytes by qRT-PCR (the peak of expression was at between 24 and 26 cycles; data not shown) and western blot analysis (Fig. 1C), respectively, and by immunocytochemistry (Fig. 1D). Its expression on the surface of human cartilage was found on immunohistochemistry (Fig. 1E). Thus, CD13 is constitutively expressed in articular chondrocytes.

### CD13 knockdown inhibits 14-3-3ε-induced MMP-3 and MMP-13 expression in murine chondrocytes

**Fig. 2.**
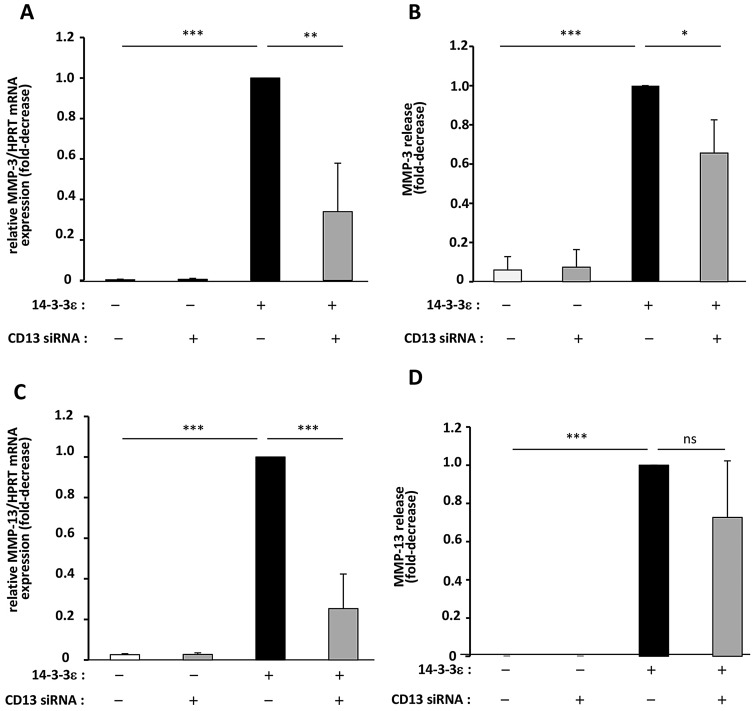
**Involvement of CD13 in 14-3-3ε-induced MMP-3 and MMP-13 release by murine articular chondrocytes.** Mouse articular chondrocytes were transfected for 48 h with small interfering (siRNA) targeting the CD13 gene or with negative control AllStars. The cells were then starved and stimulated with recombinant 14-3-3ε (1 µg/ml) for 24 h. (A,C) Total RNA was extracted and mRNA levels of MMP-3 (A) and MMP-13 (C) were determined by qRT-PCR. (B,D) Protein levels of MMP-3 (B) and MMP-13 (D) in cell supernatants were measured by ELISA. 14-3-3ε-stimulated cells released 756.68±69 ng/ml and 347.3±48 pg/ml of MMP-3 and MMP-13, respectively. Bars show the mean±s.d. fold decrease in expression compared to the negative control-transfected chondrocytes stimulated with 14-3-3ε (set at 1) from three independent experiments. Blots in D are representative of three independent experiments. *P<0.05; **P<0.01; ***P<0.001; ns, not significant.

To investigate the role of CD13 in 14-3-3ε-mediated MMP-3 and MMP-13 expression, we used siRNA-mediated knockdown. Mouse chondrocytes transfected with siRNA1 or siRNA2 against CD13 showed significantly decreased expression of CD13 expression at the mRNA level (by 42% and 59%, respectively, as compared with the negative control, *P*<0.05) and the protein level (by 79% and 88%, respectively, as compared with the negative control, *P*<0.05) (supplementary material Fig. S1), which demonstrates efficient gene silencing. We selected siRNA2 for the following experiments. Mouse chondrocytes were transfected with the negative control siRNA or CD13 siRNA2, then treated with 14-3-3ε for 24 h to examine MMP-3 and MMP-13 expression. Treatment with 1 µg/ml 14-3-3ε increased the mRNA and protein levels of MMP-3 by tenfold as compared with the control. The 14-3-3ε-mediated induction of MMP-3 expression and release was significantly inhibited by CD13 siRNA (∼66% and ∼45%, respectively, relative to stimulated controls) (*P*<0.05; Fig. 2A,B). Similarly, treatment with 14-3-3ε increased chondrocyte MMP-13 expression and secretion (tenfold and fivefold, respectively, relative to unstimulated controls). The 14-3-3ε-mediated induction of MMP-13 expression and release was significantly decreased by CD13 siRNA (∼75% and ∼23%, respectively, relative to stimulated controls) (*P*<0.01; Fig. 2C,D). Thus, CD13 knockdown strongly reduced the 14-3-3ε-induced mRNA and protein expression of both MMP-3 and MMP-13, so CD13 might be involved in establishing a catabolic phenotype in murine articular chondrocytes induced by 14-3-3ε.

### Articular chondrocytes exhibit aminopeptidase N activity not modified by 14-3-3ε

**Fig. 3.**
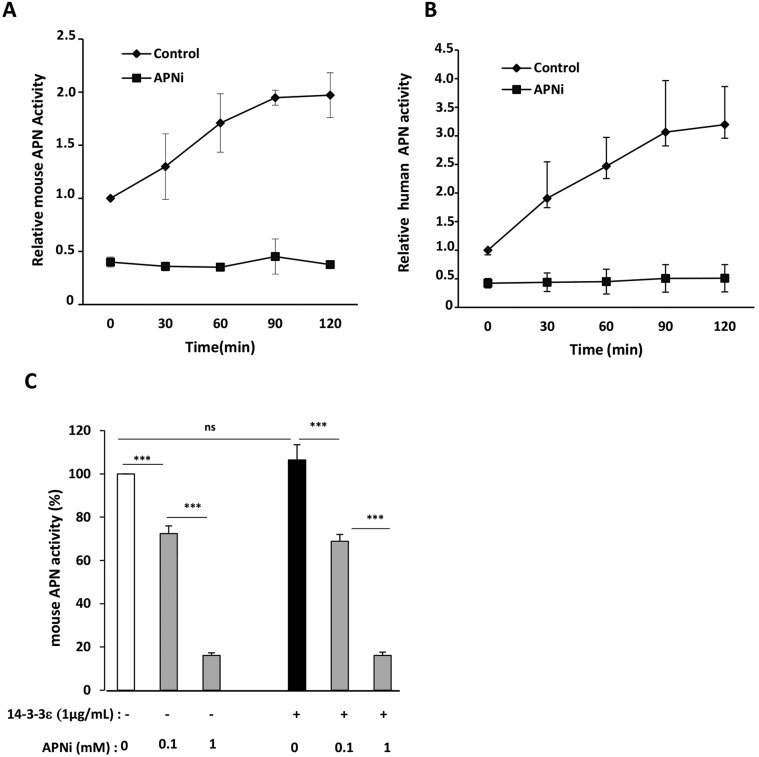
**Kinetics of enzymatic activity of APN and 14-3-3ε effect in murine and human articular chondrocytes.** Kinetics of enzymatic activity of APN in mouse (A) and human (B) articular chondrocytes. Primary cultures of articular chondrocytes were starved, then incubated with fluorescent substrate, L-alanine β-naphthylamide (50 µM), for the indicated times (0 to 120 min) in the absence (control) or in the presence of a specific aminopeptidase inhibitor (APNi) (1 mM). Data are mean±s.d. relative increase and decrease in fluorescence compared to control aminopeptidase N activity set to 1 (before adding the substrate and without APNi treatment) from three independent experiments. (C) 14-3-3ε effect on aminopeptidase N activity. Primary cultures of mouse articular chondrocytes were starved, pretreated with APNi (0.1 and 1 mM), then stimulated with 14-3-3ε (1 µg/ml). After 24 h, cells were incubated with L-alanine β-naphthylamide (50 µM) for 60 min and fluorescence was measured. Data are mean±s.d. relative decrease in fluorescence versus control aminopeptidase N activity set to 100 (without stimulation with 14-3-3ε and without APNi) from three independent experiments *P<0.05; **P<0.01; ***P<0.001; ns, not significant.

CD13 can act through its enzymatic activity. To first determine the presence of this activity in articular chondrocytes, we examined the kinetics of aminopeptidase N activity at different times using a specific substrate (L-alanine β-naphtylamide, 50 µM) with or without an aminopeptidase N inhibitor (APNi; 1 mM). In mouse chondrocytes, aminopeptidase N activity increased over time, to peak at 60–90 min (∼twofold increase versus control); this activity was strongly inhibited by APNi (Fig. 3A). To test the effect of 14-3-3ε aminopeptidase N enzymatic activity, mouse chondrocytes were stimulated with 14-3-3ε with or without APNi (0.1 and 1 mM). 14-3-3ε did not alter aminopeptidase N activity. The dose-dependent inhibitory effect of APNi on enzymatic activity was not modified by 14-3-3ε (Fig. 3C). Similar results were obtain at 60 and 90 min of 14-3-3ε stimulation (only the time 60 min is shown in Fig. 3C). In addition, human osteoarthritis articular chondrocytes showed aminopeptidase N activity, which increased over time (∼threefold increase at 90 min) and was strongly inhibited by APNi (Fig. 3B).

### 14-3-3ε invokes the receptor role of CD13 to induce MMP-3 and MMP-13 expression in chondrocytes

**Fig. 4.**
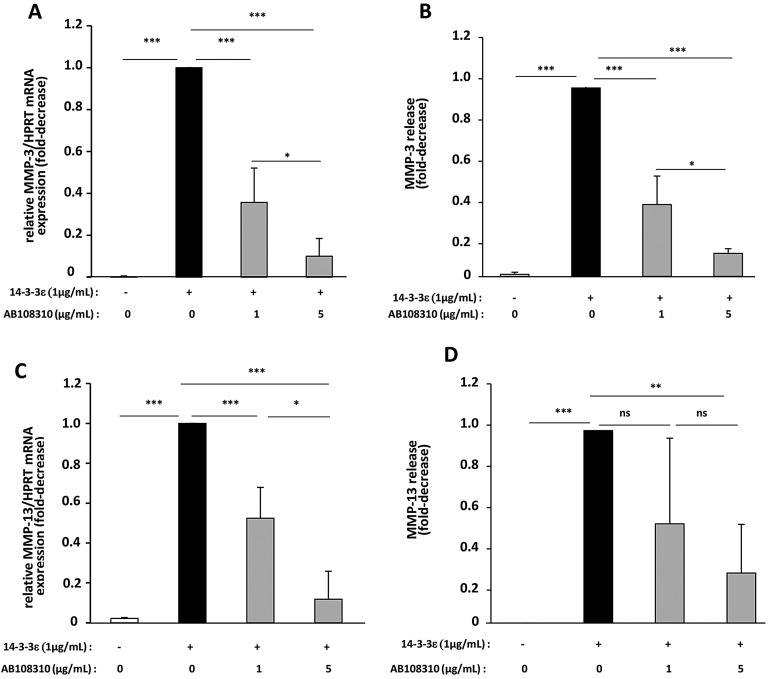
**Inhibition of the expression and release of MMP-3 and MMP-13 in murine chondrocytes by anti-CD13 blocking antibody.** Primary cultures of articular chondrocytes from newborn mice were starved, then stimulated with 1 µg/ml recombinant 14-3-3ε in the presence of an anti-CD13 blocking antibody (AB108310) at 1 or 5 µg/ml for 24 h. (A,C) Total RNA was extracted and the mRNA levels of MMP-3 (A) and MMP-13 (C) were determined by qRT-PCR. (B,D) Protein levels of MMP-3 (B) and MMP-13 (D) in cell supernatants were measured by ELISA. 14-3-3ε-stimulated cells released 621.4±50 ng/ml and 286.7±17.1 pg/ml of MMP-3 and MMP-13, respectively. Bars show the mean±s.d. fold decrease in expression compared to control cells stimulated with 14-3-3ε (set at 1) from three independent experiments. *P<0.05; **P<0.01; ***P<0.001; ns, not significant.

To investigate the involvement of the CD13 receptor in 14-3-3ε-mediated MMP-3 and MMP-13 activity, we used an anti-CD13 blocking antibody to block only the receptor role of this protein. We pretreated primary cultures of murine and human chondrocytes with or without the anti-CD13 blocking antibody, followed by stimulation with 14-3-3ε, and compared the results in untreated and stimulated chondrocytes. In murine chondrocytes, 14-3-3ε-induced MMP-3 mRNA expression was inhibited dose dependently by anti-CD13 blocking antibody (inhibition of 64% at 1 µg/ml and 90% at 5 µg/ml), as was MMP-3 protein release (inhibition of 61% at 1 µg/ml and 87% at 5 µg/ml) (*P*<0.05; Fig. 4A,B). Similarly, the blocking antibody dose dependently decreased 14-3-3ε-induced MMP-13 mRNA expression (47% at 1 µg/ml and 88% at 5 µg/ml, *P*<0.05; Fig. 4C) and MMP-13 release (29% at 1 µg/ml and 32% at 5 µg/ml, *P*>0.05; Fig. 4D). The blocking antibody alone has no effect on mouse MMP-3 and MMP-13 gene expression (data not shown).

To confirm these results in human chondrocytes, cells were pretreated with a specific human anti-CD13 blocking antibody (SJ1D1, 1 µg/ml), which is widely used to block only the receptor role of human CD13 and has no effect on its enzymatic activity. Cells were then stimulated with 14-3-3ε for 24 h. 14-3-3ε-induced MMP-3 mRNA expression and protein release were significantly decreased by 43% and 59%, respectively (*P*<0.05; supplementary material Fig. S2). As observed in previous publications, MMP-13 expression in human chondrocytes was very low. Thus, the results obtained by qRT-PCR were not interpretable. Taken together, these results show that treatment with an antibody that blocks only the receptor role of CD13 greatly inhibits the 14-3-3ε-mediated mRNA and protein expression of both MMP-3 and MMP-13 in murine and human chondrocytes, which suggests that CD13 has a receptor role in transmitting the 14-3-3ε signal in chondrocytes and inducing a procatabolic phenotype.

### 14-3-3ε interacts with CD13

**Fig. 5.**
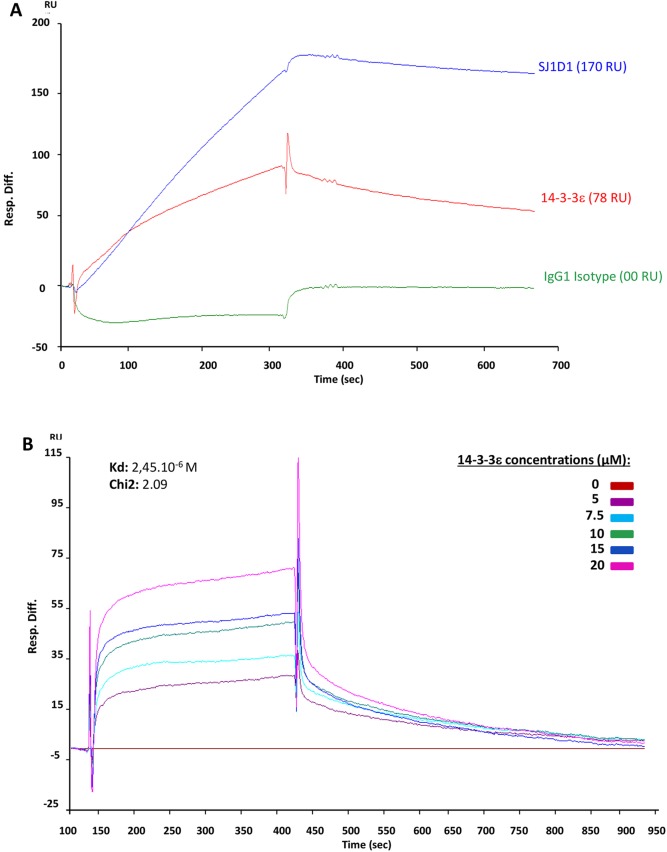
**Interaction between 14-3-3ε and CD13.** (A) Representative SPR sensorgrams showing the binding of recombinant human 14-3-3ε (red, 10 µM), human anti-CD13 antibody SJ1D1 (blue, 1 µg/ml), and isotype IgG1 control (green, 1 µg/ml) on recombinant human CD13 immobilized on the sensor chip. The differential response was obtained by subtracting the signal in the blank channel from that in the experimental channel. Response is expressed in resonance units (RU) relative to time in seconds. (B) Kinetics of 14-3-3ε binding to CD13. Six concentrations of 14-3-3ε (5, 7.5, 10, 15 and 20 µM) were tested. The base line (in red) corresponds to HBS-EP alone. Software analysis gave a *K*_D_ value of 2.45×10^−6^ M with χ-squared value 2.09.

To test whether 14-3-3ε interacts directly with CD13, we performed surface plasmon resonance (SPR) experiments. Human recombinant CD13 was covalently immobilized on a CM5 chip. Human recombinant 14-3-3ε, a specific blocking antibody against human CD13 and an IgG1 isotype control were used as analytes. Results showed a direct interaction between recombinant CD13 and 14-3-3ε [78 resonance units (RU)], with higher binding with blocking antibody (170 RU) and no binding with IgG1 isotype control (00 RU) (Fig. 5A). Kinetic tests with increasing concentrations of recombinant 14-3-3ε showed that 14-3-3ε bound with low affinity to CD13, with a dissociation constant (*K*_D_) of 2.45×10^−6^ M (χ-square value 2.09, showing good fit) (Fig. 5B). These results suggest that CD13 interacts directly with extracellular 14-3-3ε to transmit its signal in chondrocytes.

### 14-3-3ε binds to the cell surface

**Fig. 6.**
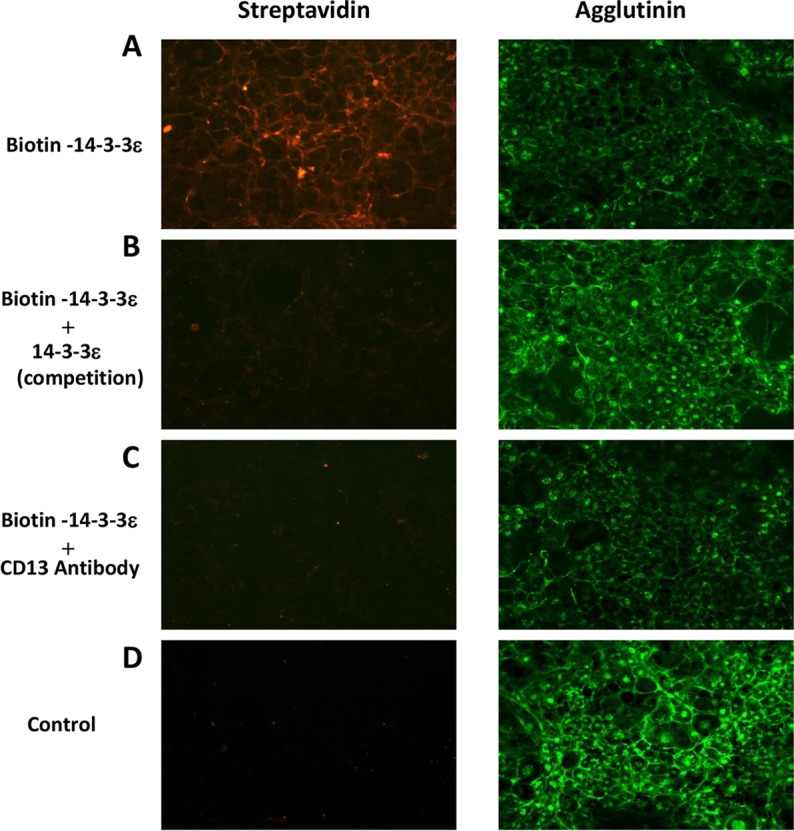
**14-3-3ε binding to the chondrocyte surface.** Mouse chondrocytes were treated with biotin–14-3-3ε for 15 min in the absence (A) or in presence of excess 14-3-3ε (B) or anti-CD13 blocking antibody (AB108310; Abcam) (C). Cells not treated with biotin–14-3-3ε were used as a negative control (D). Cells were analyzed by immunofluorescence microscopy, with Alexa-Fluor-568-conjugated streptavidin or Alexa-Fluor-488-conjugated agglutinin as the plasma membrane marker.

We assessed the ability of 14-3-3ε to bind to the surface of mouse chondrocytes by incubating these cells with biotin-labeled recombinant 14-3-3ε. The cells were then incubated with fluorescently labeled streptavidin and analyzed by fluorescence microscopy. Biotin–14-3-3ε bound to the chondrocyte surface (Fig. 6A). We performed a competition assay, in which cells were incubated with excess unlabeled 14-3-3ε before the addition of biotin–14-3-3ε. The unlabeled competitor strongly decreased biotin–14-3-3ε binding to the chondrocyte surface (Fig. 6B), thereby demonstrating the specificity of binding. We investigated the involvement of CD13 in biotin–14-3-3ε binding to cells, by subjecting the cells to prior treatment with anti-CD13 blocking antibody (AB108310, 5 µg/ml). Biotin–14-3-3ε binding was decreased by CD13 blocking (Fig. 6C). No cell binding signal was detected in control cells (Fig. 6D). These results therefore confirm that 14-3-3ε–CD13 interactions probably occur on the surface of chondrocytes.

### E^579^FNYVW^584^ is the potential CD13-binding site in 14-3-3ε

**Fig. 7.**
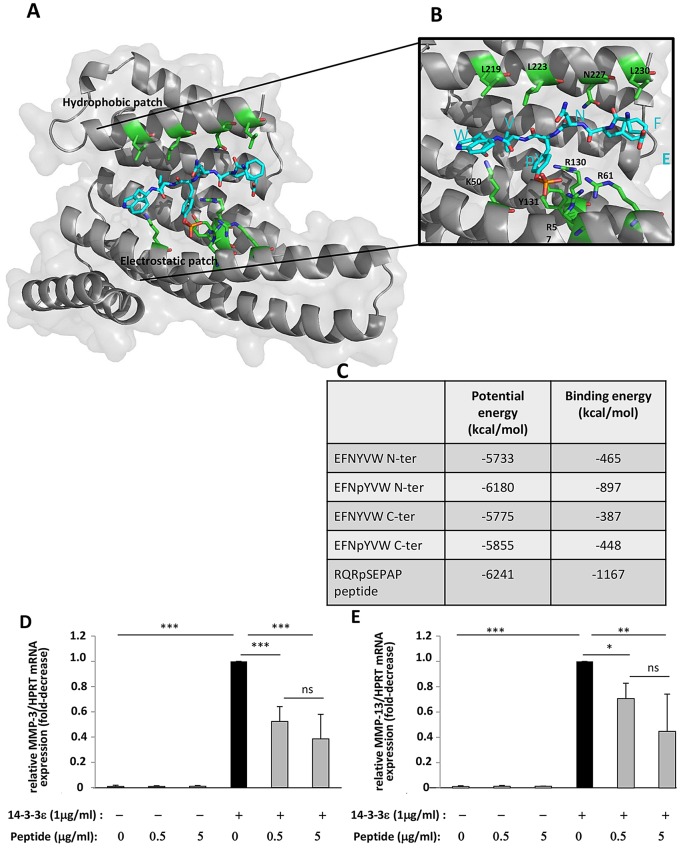
**EFNpYVW is probably the amino acid sequence of CD13 recognized by 14-3-3ε.** (A) 14-3-3ε is shown as a gray ribbon and EFNpYVW as blue sticks with the phosphorylated Tyr 582 highlighted (in orange sticks). The residues of 14-3-3ε involved in the binding are shown as green sticks. (B) Higher magnification of the ligand-binding channel with the amino acid residues involved in the binding of EFNpYVW. Structural images were generated by use of PyMOL (www.pymol.org). (C) Data table of energy binding parameters for the reference RQRSAPAP, EFNYVW and EFNpYVW peptides, positioned as the reference (N-ter) or in the reverse orientation (C-ter). (D,E) Primary cultures of mouse articular chondrocytes were serum starved, and incubated with various concentrations (0, 0.5 and 5 µg/ml) of EFNpYVW peptide. They were then unstimulated or were stimulated with 1 µg/ml recombinant 14-3-3ε for 24 h. Total RNA was extracted and the levels of mRNA for MMP-3 (D) and MMP-13 (E) were determined by qRT-PCR. Bars show the mean±s.d. fold decrease in expression with respect to control cells stimulated with 14-3-3ε (set to 1), for three independent experiments. *P<0.05; **P<0.01; ***P<0.001; ns, not significant.

To identify the putative motif(s) on CD13 that could bind to 14-3-3ε, we examined the sequence and structure of CD13 (PDB ID 4FYQ) (Wong et al., 2012). We defined three criteria to identify potential binding site(s). First, we looked for phosphorylated residues, with a preference for a phosphorylated Tyr because previous work has shown that 14-3-3ε recognizes phosphorylated Ser, Thr or Tyr residues with a marked preference for Tyr (Uhart and Bustos, 2013, 2014). Second, the phosphorylated motif(s) had to be located at a position on the structure of the CD13 protein where they would be accessible to phosphorylation by a Ser, Thr or Tyr kinase, and third, they should belong to a segment able to adopt an extended conformation, because all ligands bound to 14-3-3ε display an elongated binding mode (Yang et al., 2006; Bustos and Iglesias, 2006). Tyr582 was identified to have these criteria among several other Ser and Thr residues. This unique Tyr residue of the mature protein has been shown to be phosphorylated *in vivo* (Hornbeck et al., 2012) and it is the only accessible phosphorylatable residue, as evidenced by the structure of CD13. TyrY582 is part of a long, accessible and elongated loop. In agreement, Y582 inserted in the sequence E_579_FNYVW_584_ is flanked by charged or polarized residues that are compatible with the preferred recognition sequence of 14-3-3ε, RSXpSXP (mode 1), where pS is a phosphorylated Ser residue (Obenauer et al., 2003; Yaffe et al., 1997). Y582 was selected for further docking calculations. The EFNYVW was docked in the binding groove of 14-3-3ε and positioned by analogy with the hexapeptide RQRpSAP complexed to 14-3-3ε. Two orientations (denoted N-ter and C-ter) and two phosphorylation states (phosphorylated and non-phosphorylated) were tested and compared to the crystal complex. The docking of EFNpYVW with the best scoring energies was found to be oriented in a similar position to that of the solved RQRpSAP ligand, which served as a reference. This complex showed both the lowest total potential energy and the best interaction energy with the protein 14-3-3ε (−897 and −6180 kcal/mol, respectively) (Fig. 7C). In addition, residues of 14-3-3ε, involved in the binding of the crystal peptide, were identified to be involved in the binding of the CD13 hexapeptide fragment. Of note, the groove shows a hydrophobic patch whereby L219, L223 and L230 interact with the hydrophobic facet of the ligand peptide. On the other side of its groove, 14-3-3ε shows a heavily charged region, composed of K50, K57, R61, R130 and Y131, that is particularly able to accommodate the negatively charged phosphorylated Tyr (Fig. 7A,B). Taken together, these results suggest that the 14-3-3ε can accommodate the segment E^579^FNYVW^584^ of CD13. Nonetheless, larger conformational changes for CD13 and probably for 14-3-3ε should be investigated to reveal better recognition between the two proteins. Such molecular deciphering at this stage is too unreliable to be computed *in silico*. Nevertheless, Y582 inserted in the segment, E^579^FNpYVW^584^, is a good phosphorylation candidate for 14-3-3ε recognition and binding.

To confirm our *in silico* analysis and assess the involvement of E^579^FNYVW^584^ as the binding motif between CD13 and 14-3-3ε, mouse chondrocytes were subjected to preincubation with EFNpYVW and were then stimulated with 14-3-3ε in the presence or absence of EFNpYVW, to prevent binding to endogenous CD13. 14-3-3ε-induced MMP-3 mRNA production was dose dependently inhibited by the peptide (48% inhibition at 0.5 µg/ml and 62% at 5 µg/ml *P*<0.05; Fig. 7D). Similarly, EFNpYVW dose dependently decreased 14-3-3ε-induced MMP-13 mRNA synthesis (by 30% at 0.5 µg/ml and 56% at 5 µg/ml, *P*<0.05; Fig. 7E). This suggests that EFNpYVW is the peptide sequence within CD13 required for the recognition of 14-3-3ε.

## DISCUSSION

To induce the chondrocyte procatabolic phenotype, extracellular 14-3-3ε must bind to a receptor expressed on the surface of articular chondrocytes. The aim of this study was to investigate the involvement of CD13, the supposed receptor for 14-3-3ε, in bone–cartilage communication. Through its enzymatic activity, CD13 is able to modulate the activity of numerous peptides that participate in important biological processes such as components of extracellular matrix (Menrad et al., 1993; Saiki et al., 1993) and neuropeptides (Matsas et al., 1985; Jia et al., 2010). CD13 is also a receptor for human coronavirus (Kolb et al., 1998) and cytomegalovirus (Söderberg et al., 1993). Signal transduction has recently been proposed as also being one of the CD13 functions that are independent of its enzymatic activity (Mina-Osorio, 2008).

We found that 14-3-3ε-mediated MMP-3 and MMP-13 expression was suppressed in murine chondrocytes transfected with CD13 siRNA, which suggests the involvement of CD13 in the cellular effects of 14-3-3ε .

As we mentioned previously, CD13 performs its functions through its receptor role and/or its aminopeptidase N enzymatic activity. Several inhibitors of the aminopeptidase N activity were synthesized (Piedfer et al., 2011). We chose an APNi, 2′-3-dinitriflavone-8-acetic-acid, to verify the specificity of this activity in articular chondrocytes. In our experiments, the doses of APNi were adjusted according to the literature and cytotoxicity values obtained after lactate dehydrogenase assay in supernatants of treated chondrocytes. First, we demonstrated the presence of aminopeptidase N activity in articular chondrocytes by using the fluorescent substrate L-alanine β-naphthylamide. This aminopeptidase N activity was strongly inhibited in chondrocytes in the presence of APNi but not altered after stimulation with 14-3-3ε. These results suggest that the active site of the enzyme might be distinct from the binding site of 14-3-3ε on CD13. To confirm that, we tested the effect of APNi on 14-3-3ε-mediated MMP-3 and MMP-13 expression. A previous study (Ghaffari et al., 2010) has shown that bestatin (a potent inhibitor of the aminopeptidase N activity) had no effect on the 14-3-3σ-mediated MMP-1 expression. However, our results showed that APNi, used to inhibit the enzyme activity, inhibited also MMP-3 and MMP-13 expression induced by 14-3-3ε (data not shown). This effect could result from a change in CD13 conformation induced by the binding of the inhibitor to the active site. As a result, 14-3-3ε could be unable to bind to its binding domain (which would become concealed) to induce the expression of MMP-3 and MMP-13. This hypothesis is based on a study (Chen et al., 2012) showing that the binding of a peptide to the active site leads CD13 to convert from an open inactive state to a closed active state.

We found that the anti-CD13 blocking antibody AB108310 for murine chondrocytes and SJ1D1 for osteoarthritis human chondrocytes, inhibited the chondrocyte response to recombinant 14-3-3ε by decreasing the expression and release of MMP-3 and MMP-13. These antibodies were tested for specifically recognizing CD13 by western blot analysis. Preliminary experiments showed that AB108310 did not alter the aminopeptidase N enzymatic activity. Similarly, SJ1D1 was shown to be specific to human CD13 without affecting the aminopeptidase enzymatic activity (Piedfer et al., 2011). Thus, the catalytic site is not involved in the inhibitory effect of the anti-CD13 blocking antibody. The decrease in expression and release of MMP-3 and MMP-13, despite stimulation with 14-3-3ε, was indeed due to deletion of the receptor role of CD13 but not its enzymatic activity. Growing evidence suggests that CD13 might have an array of functions that are independent of its enzymatic activity, including its role as a viral receptor (Wong et al., 2012). Similarly, human monocytic aggregation depends on CD13 signal transduction but is independent of its enzymatic activity (Delmas et al., 1994).

By SPR analysis, we found that 14-3-3ε binds directly to CD13. This technique has been found to be effective in the analysis of direct interaction between proteins. The specific interaction between 14-3-3ε and CD13 was evidenced by SPR with human recombinant CD13 covalently bound on a chip and injected human recombinant 14-3-3ε. Injection of specific blocking antibody against CD13, and IgG isotype negative and positive controls, demonstrated that the interaction between 14-3-3ε and CD13 was specific. This direct interaction was confirmed by kinetic tests, although with weaker binding affinity (*K*_D_=2.45×10^−6^ M), which might be explained by the absence of some amino acid sequences from recombinant 14-3-3ε and CD13 used in SPR analysis. Using labeled biotin–14-3-3ε, we showed that 14-3-3ε binds to the surface of chondrocytes in a manner that is dependent on CD13. Taken together, these results confirm the potential involvement of CD13 in 14-3-3ε signal transmission in chondrocytes and strongly suggest that direct interaction between 14-3-3ε and CD13 could occur in bone–cartilage communication.

However, the mode of binding of 14-3-3ε to CD13 has not been determined. Therefore, we investigated the potential 14-3-3ε-binding site in CD13. The hexapeptide E^579^FNpYVW^584^ shows an energy of interaction with 14-3-3ε in the same range as that displayed by RQRpSAP ligand to 14-3-3ε in the crystal complex. Indeed, among the variants tested, this ligand shows the highest affinity to 14-3-3ε in terms of interaction energy computed, that is, when the Tyr582 was phosphorylated. In addition, this segment belongs to an unfolded and accessible loop in CD13. Of note, this region of CD13 is likely more accessible in its open state, as described previously (Mina-Osorio et al., 2006) and modeled by us (data not shown). 14-3-3 proteins can distinguish between phosphorylated and non-phosphorylated partners (Bustos, 2012), and many cellular processes are regulated by the binding of 14-3-3 to phosphorylated sites in target proteins (Yasmin et al., 2010). Accordingly, our experimental data reveal an interaction between CD13 and 14-3-3ε domains, previously predicted by our *in silico* studies that identify Y582 as a good candidate for phospho-modification. Such binding of 14-3-3ε to phosphorylated CD13 strongly supports the idea that phosphorylation might regulate CD13 signaling. Moreover, pre-incubation of cells with the mimic peptide EFNpYVW, identified in CD13, which contains a phosphorylation site at Y582, prevents 14-3-3ε binding to CD13 to induce its catabolic effect. This experiment validates candidate EFNpYVW as the CD13 peptide motif involved in 14-3-3ε recognition and binding.

Concerning cell signaling pathways involved in 14-3-3 signal transmission, our results show that specific inhibitors of p38 MAPKs and JNK inhibit MMP-3 and MMP-13 expression in response to 14-3-3ε in articular chondrocytes (supplementary material Fig. S3). However, no effect of ERK inhibitor on chondrocyte response to 14-3-3ε was found (supplementary material Fig. S3). Some published reports have demonstrated a link between 14-3-3 proteins and MAPK signaling cascades. It has recently been suggested that the stimulation of cells with 14-3-3η leads to the phosphorylation of ERK and JNK, but not p38 MAPKs, inducing mediators of inflammation and joint destruction in rheumatoid arthritis (Maksymowych et al., 2014). Lam and colleagues have also reported that 14-3-3σ-induced fibroblast MMP-1 expression was mediated through p38 MAPKs and upregulated c-Jun and c-Fos expression (Lam et al., 2005). In addition, CD13 knockdown could block activation of p38 MAPKs in response to 14-3-3σ stimulation in fibroblasts (Ghaffari et al., 2010). However, anti-CD13 blocking antibodies can inhibit ERK1/2, JNK and p38 phosphorylation (Santos et al., 2000), which suggests a link between CD13 and MAPK pathways. A role for CD13 in signal transduction, independent of its enzymatic activity, has been reported in monocytic cell adhesion (Delmas et al., 1994). However, CD13 possesses a short intracellular domain (seven amino acids) without a signaling motif. Thus, CD13 needs to associate with other proteins to initiate a signaling cascade. Many proteins are found in complex with CD13 including galectin-3, Grb2, Sos, galectin-4 and reversion-inducing cysteine-rich protein with kazal motifs (Luan and Xu, 2007). Moreover, Mina-Osorio and colleagues have shown that CD13 interacts with IgG receptors (FcγRs) on the surface of monocytic cells (Mina-Osorio and Ortega, 2005), so CD13 transduction might be activated by other receptors.

We used human osteoarthritis joint samples to strengthen the concept that CD13 is involved in establishing the procatabolic phenotype induced by 14-3-3ε in chondrocytes. The results validated our findings in murine chondrocytes and confirmed that CD13 might play a role in osteoarthritis bone–cartilage communication by transmitting the 14-3-3ε signal in chondrocytes. We identify a direct interaction between CD13 and 14-3-3ε, which suggests that CD13 might be a cell surface receptor or co-receptor for 14-3-3ε. The 14-3-3ε and CD13 interaction could represent a new therapeutic target in osteoarthritis.

## MATERIALS AND METHODS

### Materials

All reagents were purchased from Sigma-Aldrich (Lyon, France), unless stated otherwise. Fetal bovine serum (FBS) was obtained from Invitrogen (Cergy-Pontoise, France). Liberase and complete protease inhibitor mixture were from Roche Diagnostics (Meylan, France). The enhanced chemiluminescence western blot analysis kit was from Amersham Pharmacia Biotech (Orsay, France). The immunoblot polyvinylidene difluoride membranes for western blot analysis and kaleidoscope prestained standards were from Bio-Rad (Marnes-la-coquette, France). Recombinant human 14-3-3ε was from Enzo Life Sciences.

### Primary culture and stimulation of human articular chondrocytes

Human cartilage samples were obtained from osteoarthritis patients undergoing total joint replacement surgery for osteoarthritis at Saint-Antoine Hospital (Paris, France). These materials are considered surgical waste and are used with informed consent and in accordance with French ethics laws (L.1211-2 to 1211-7, L.1235.2, and L.1245.2). Investigations conformed to the principles outlined in the Declaration of Helsinki. The osteoarthritis diagnosis was based on clinical and radiographic evaluations according to the American College of Rheumatology criteria (American College of Rheumatology Subcommittee on Osteoarthritis Guidelines, 2000).

Cartilage samples were obtained from the tibial plateau and femoral condyle of osteoarthritis patients, cut into small pieces (∼1 mm^3^), and washed several times with phosphate-buffered saline (PBS). All enzymatic digestions were performed at 37°C, under agitation, in Dulbecco's modified Eagle's medium (DMEM) containing 4.5 g/l glucose, 100 units/ml penicillin, 100 µg/ml streptomycin and 4 mM glutamine (Sigma-Aldrich, Saint Quentin Fallavier, France). Samples were incubated twice for 45 min each, in 25 ml Liberase (Roche Diagnostics) at 0.52 Wünsch units (UW)/ml to eliminate surrounding extracellular matrix, then incubated overnight with 25 ml Liberase at 0.13 UW/ml. The cell suspension was filtered through a 100-µm cell strainer and centrifuged for 6 min at 400 ***g***. Chondrocytes were cultured for 8 to 10 days in 12-well plates (250,000 cells/well) in DMEM containing 4.5 mg/l glucose, 100 units/ml penicillin, 100 µg/ml streptomycin and 4 mM glutamine, supplemented with 15% FBS and allowed to grow to confluence. Cells were then starved in serum-free medium containing 0.1% bovine serum albumin (BSA) for 24 h. For blocking antibody experiments, chondrocytes were treated for 15 min with SJ1D1 (1 µg/ml), a human anti-CD13 antibody (Santa Cruz Biotechnology), before stimulation with recombinant 14-3-3ε (1 µg/ml).

### Primary culture and stimulation of murine articular chondrocytes

All experiments followed protocols approved by the French or European ethics committee. Mouse articular chondrocytes were isolated by enzymatic digestion of articular cartilage from C57BL/6J mice (5–6 days old), as described previously (Salvat et al., 2005). After 6 to 7 days of culture, chondrocytes were placed in DMEM (1 g/ml glucose) containing 100 units/ml penicillin, 100 µg/ml streptomycin, and 4 mM glutamine and supplemented with 0.1% BSA for 24 h. For blocking antibody experiments, chondrocytes were treated for 15 min with increasing concentrations (1 and 5 µg/ml) of a mouse anti-CD13 antibody (AB108310; Abcam), then stimulated with recombinant 14-3-3ε (1 µg/ml) for 24 h. For peptide experiments, murine chondrocytes were treated for 15 min with two concentrations (0.5 and 5 µg/ml) of peptide solution (EFNpYVW, 96.04% pure, Proteogenix), then stimulated with recombinant 14-3-3ε (1 µg/ml) for 24 h. For the inhibition of MAPK pathways, cells were incubated for 15 min with two concentrations (5 and 25 µM) of MAPK-specific inhibitors (SB203580 for p38 MAPKs, PD98059 for ERK1/2 and JNK inhibitor II for JNK; Merck Millipore) and then stimulated with recombinant 14-3-3ε (1 µg/ml) for 24 h

### Immunohistochemical analysis

Human cartilage samples were fixed in 4% paraformaldehyde for 72 h before paraffin embedding. Transverse sections (4 µm thick) were cut parallel to the rib axis by use of a Polycut E microtome (Leica, Wetzlar, Germany), then mounted on slides. For labeling, slides were deparaffinized and unmasked by use of citrate buffer (pH 6) at 95°C, then blocked in a solution of TBS-TC [0.745 g/l Tris-HCl, 9 g/l NaCl, 0.02% Tween 20, 0.6 g/l caseine (Sigma), pH 7.4]. Human CD13 was detected with monoclonal rabbit antibody (AB108310; Abcam). Rabbit IgG (DAKO) was a negative control. Secondary antibody and peroxidase were added by use of the horseradish-perioxidase-conjugated LSAB+ system (DAKO). Visualization involved the DAB kit (VECTOR). Image-Pro express was used for image capture.

### Immunocytochemical analysis

Mouse and human chondrocytes were fixed in 4% paraformaldehyde, permeabilized (0.1% Triton X-100), then blocked in a solution of TBS-TC. Cells were exposed to anti-CD13 antibody (AB108310; Abcam) or an isotype control antibody (rabbit IgG; DAKO). Secondary antibody and peroxidase were added by use of the horseradish perioxidase-conjugated LSAB+ system (DAKO). Visualization involved the DAB kit (VECTOR). Image-Pro express was used for image capture.

### siRNA knockdown of CD13

Mouse CD13 knockdown was tested with two different siRNA oligonucleotide sequences: si-CD13-1, 5′-ATGGATCTTACTGAACATTAA-3′, and si-CD13-2, 5′-CCGGGTGATCCTGAGACCCTA-3′ (Qiagen). AllStars was a negative control. Mouse chondrocytes were cultured as described above. Confluent cells were removed with a mixture of collagenase P and pronase. Cells were centrifuged (1600 rpm for 6 min) and the pellet was resuspended in PBS (Sigma-Aldrich).

Murine articular chondrocytes were transfected by use of an electroporation device (Amaxa) according to the manufacturer's instructions (Qiagen). Briefly, cells (4×10^6^) were resuspended in Amaxa electroporation transfection solution with siRNA (1 µM), then placed in cell culture medium DMEM (1 g/l glucose) supplemented with penicillin, streptomycin and L-glutamine containing 10% FBS. At 48 h post-transfection, cells were starved in serum-free medium containing 0.1% BSA overnight, then stimulated with recombinant 14-3-3ε (1 µg/ml) for 24 h.

### RNA extraction and qRT-PCR

Total RNA was extracted from chondrocytes by use of the RNeasy Mini kit (Qiagen). Total RNA (0.5 µg) was reverse transcribed with use of the Omniscript RT kit (Qiagen). Relative quantification of genes involved the Light Cycler 480 Real-Time PCR Detection System (Roche Diagnostics) and GoTaq qPCR Master Mix (Promega) as described previously (Blaise et al., 2009).

For amplification of mouse cDNA, oligonucleotide primer sequences were for MMP-3, sense 5′-TGAAAATGAAGGGTCTTCCGG-3′ and anti-sense 5′-GCAGAAGCTCCATACCAGCA-3′; MMP-13, sense 5′-GATGGCACTGCTGACATCAT-3′ and anti-sense 5′-TGTAGCCTTTGGAACTGCTT-3′; CD13, sense 5′-AATCTCATCCAGGGAGTGACC-3′ and anti-sense 5′-GTGGCTGAGTTATCCGCTTT-5′; and hypoxanthine guanine phosphoribosyltransferase (HPRT), sense 5′-AGGACCTCTCGAAGTGT-3′ and anti-sense 5′-ATTCAAATCCCTGAAGTACTCAT-3′. The relative mRNA expression of MMP-3, MMP-13 and CD13 were normalized to that of HPRT, as an internal gene reference.

For amplification of human cDNA, oligonucleotide primer sequences were for MMP-3, sense 5′-ATGAAAATGAAGGCTCTTCCG-3′ and anti-sense 5′-GCAGAAGCTCCATACCAGCA-3′, and human 18S, sense 5′-GCAATTATTCCCCATGAACG-3′ and anti-sense 5′-GGGACTTAATCAACGCAAGC-3′. The relative mRNA expression of MMP-3 was normalized to that of 18S, as an internal gene reference.

### Protein extraction and western blotting

Chondrocytes were lysed in RIPA buffer [50 mM Tris-HCl pH 8, 150 mM NaCl, 2 mM EDTA, 1% NP-40, 0.5% sodium deoxycholate, 0.1% SDS and 1× protease inhibitor cocktail (Roche Diagnostics)]. Proteins were separated on Citerion XT 4-12% Bis-Tris gels (Bio-Rad, Munich, Germany) and transferred onto nitrocellulose membranes, which were incubated with antibodies for human or mouse CD13 (Abcam) and β-actin (Sigma Aldrich) by use of the Immun-Star Western C chemiluminescence kit (Bio-Rad). For densitometry, we used Image Gauge software version 3.0 (Fujifilm).

### ELISA

Total mouse and human MMP-3 secretion was assayed in cell-free supernatants using an enzyme linked immunosorbent assay (ELISA) kit (R&D Systems) according to manufacturer's instructions. MMP-13 was also quantified in culture supernatants from murine chondrocytes using an ELISA kit as recommended by the manufacturer (Uscn Life Science Inc.). MMP-3 and MMP-13 concentrations were analyzed in duplicate at serial dilutions and were read against a standard curve.

### Assay of aminopeptidase N activity

The activity of aminopeptidase N on cultured human and mouse chondrocytes was determined according to previously published methods (Sihn et al., 2006). Briefly, cells were pretreated or not with a specific aminopeptidase inhibitor (APNi; 2′-3-dinitriflavone-8-acetic-acid, 1 mM) for 15 min, then incubated for various times (15, 30, 60, 90 and 120 min, 37°C) with 50 µM APN substrate: L-alanine β-naphthylamide (Sigma-Aldrich) in dimethyl sulfoxide (DMSO). The reaction was stopped at different times and fluorescence (emission wavelength, 330 nm; excitation wavelength, 460 nm) was measured in the supernatant by spectrofluorescence (Fluostar Galaxy). All assays were performed in duplicate.

To test the effect of 14-3-3ε on aminopeptidase N activity, cells were incubated or not with APNi for 15 min, then stimulated with recombinant 14-3-3ε (1 µg/ml). At 24 h, cells were incubated with the substrate at 37°C for 1 h. The supernatant from each well was collected and enzyme activity was determined as described above.

### SPR analysis

The interaction of 14-3-3ε and CD13 was measured using SPR on a Biacore 3000 instrument (GE Healthcare, Uppsala, Sweden) in the molecular interactions facility (IBPS, UPMC, Paris 6). Human recombinant CD13 (Sino Biological) was immobilized to a CM5 sensor chip (GE Healthcare) at 11,949 resonance units (RU) by amine coupling in 10 mM sodium acetate (pH 5.0) according to the manufacturer's instructions. 14-3-3ε (10 µM), a human CD13 antibody (SJ1D1, 1 µg/ml) and an isotype antibody (IgG1, 1 µg/ml) were diluted in HBS-EP running buffer (10 mM HEPES pH 7.4, 150 mM NaCl, 3 mM EDTA, 0.005% P20 surfactant) (GE Healthcare), then injected over the chip at a flow rate of 5 µl/min for 5 min and dissociated for 8 min. The surface of the sensor chip was regenerated by injecting 10 mM glycine-HCl, 10 mM, pH 2.0 at a flow rate of 30 µl/min for 30 s. For kinetics study, concentrations (0, 5, 7.5, 10, 15, 20 µM) of 14-3-3ε were injected. Identical injections were performed in parallel over a blank surface, at 0 RU. Data were analyzed and dissociation rate constant (K_D_) was determined by use of BIAevaluation software version 4.1. Binding and kinetics tests were first performed on empty surfaces. Data resulted from subtraction of empty-surface RU from active-surface RU.

### 14-3-3ε cell-binding analysis

Recombinant 14-3-3ε (200 µg) in PBS was labeled with a 50-fold molar excess of Sulfo-NHS-LC-Biotin (Thermo Scientific) reagent, as recommended by the manufacturer. Primary murine chondrocytes (35×10^3^) were cultured on coverslips in a 24-well plate. After six to seven days of culture, cells were placed in culture medium supplemented with 0.1% BSA for 3 h, treated with biotin–14-3-3ε for 15 min at 4°C and washed extensively with PBS, as previously described (Ghaffari, et al., 2010). For the competition assay and CD13 blocking, cells were subjected to preincubation with 200 µg/ml of 14-3-3ε for 30 min or with anti-CD13 rabbit antibody (AB108310; Abcam) for 15 min at 4°C before the addition of biotin–14-3-3ε. Finally, cells were incubated with streptavidin–Alexa-Fluor-568 (Invitrogen) and then analyzed by immunofluorescence microscopy.

### Docking of the EFNpYVW sequence of CD13 in the ligand-binding channel of 14-3-3ε

To obtain the phospho-modified sites of the human CD13, its fasta sequence P15144 was browsed in PhosphoSitePlus (http://www.phosphosite.org) (Hornbeck et al., 2012). The residues defined as phospho-modified were all mapped on the mature protein (to discard Y6 from the signal peptide), downloaded from the RCSB database (http://www.rcsb.org) under the PDB ID code 4FYQ and visualized with PyMOL Molecular Graphics System v1.5 (Schrödinger). Y582 was the unique residue inserted into an accessible and extended 20-residue-long loop. Y582 belongs to the hexamer sequence E^579^FNYVW^584^ selected for docking, to assess its capacity to accommodate into the groove of one monomer of 14-3-3ε. Chain A of the solved structure of 14-3-3ε in complex with the phosphorylated RQRpSAP ligand (PDB ID 2BR9) served as the starting coordinate. The missing atoms of K50 side-chain (lacking electronic density) were completed and the ligand was mutated into EFNYVW by use of the Build module in DiscoveryStudio, Accelrys. Two orientation modes were tested: the N-ter similar to the crystallized ligand N-ter, and the reverse C-ter. In addition, the ligands phosphorylated and not phosphorylated at Y582 were built. All these variants plus the crystal structure of chain A with RQRpSAP, as a reference, were minimized for 5000 iterations by use of the CHARMM force field (Brooks et al., 2009) and the ‘smart algorithm’ of the energy minimization module in DiscoveryStudio, Accelrys. Then, the energy interaction between the ligand and protein were evaluated in the optimized complexes by use of the interaction energy module in DiscoveryStudio. The analysis of the complexes and images involved use of PyMOL Molecular Graphics System v1.5.

### Statistical analysis

Data are expressed as the mean±s.d. relative induction compared with the control (set to 1). Data are reported as mean±s.d. unless stated otherwise. Statistical analysis was performed with the paired Student's *t*-test to compare mean values between two groups. One-way analysis of variance (ANOVA) with Turkey Kramer multiple comparisons test was used to compare mean values using GraphPad Prism software (GraphPad Software, San Diego, CA). *P*<0.05 was considered statistically significant.

## Supplementary Material

Supplementary Material
