# Supplementary material for: The pro-inflammatory cytokine 14-3-3ε is a ligand of CD13 in cartilage
Source: J Cell Sci. 2015 Sep 1;128(17):3250–62. doi: 10.1242/jcs.169573 (PMC4582189; doi:10.1242/jcs.169573)
Supplement: Supplementary Material [file supp_128_17_3250__index.html]

The pro-inflammatory cytokine 14-3-3ε is a ligand of CD13 in cartilage — Supplementary Material 

# The pro-inflammatory cytokine 14-3-3ε is a ligand of CD13 in cartilage

## JCS169573 Supplementary Material

- Supplementary Material
