# Supplementary material for: The pro-inflammatory cytokine 14-3-3ε is a ligand of CD13 in cartilage
Source: J Cell Sci. 2015 Sep 1;128(17):3250–62. doi: 10.1242/jcs.169573 (PMC4582189; doi:10.1242/jcs.169573)
Supplement: Supplementary Material [file supp_jcs.169573_JCS169573supp.pdf]

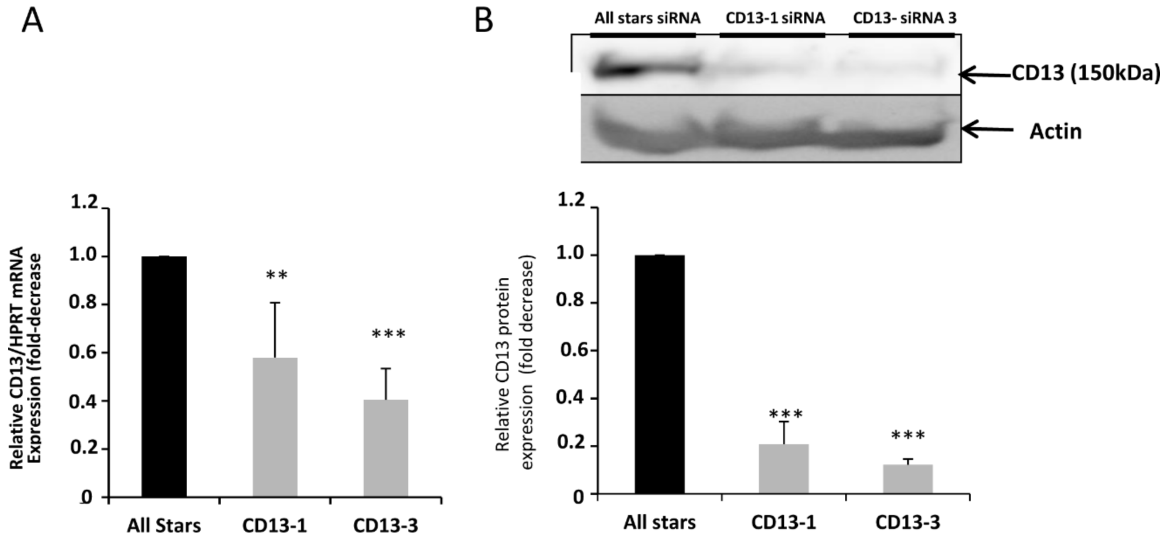

Figure S1 : Efficiency of CD13/APN siRNA. A. mRNA expression of CD13/APN in murine articular chondrocytes transfected with 2 different set of small interfering RNA (siRNA) named CD13-1 and CD13-2, targeting the CD13/APN gene, or with negative control siRNA (All stars). mRNA expression was analyzed using real-time RT-PCR to confirm the knock-down of CD13/APN expression. **B.** Protein expression of CD13/APN in murine articular chondrocytes transfected with small interfering RNA (siRNA) targeting the CD13 gene, or with negative control siRNA (All stars). Protein expression was analyzed using immunoblot to confirm the knock-down of CD13/APN expression. Blots are representative of three independent experiments. Data are mean±SD relative decrease in CD13/APN expression from three independent experiments. **p<0.01; ***p<0.001.

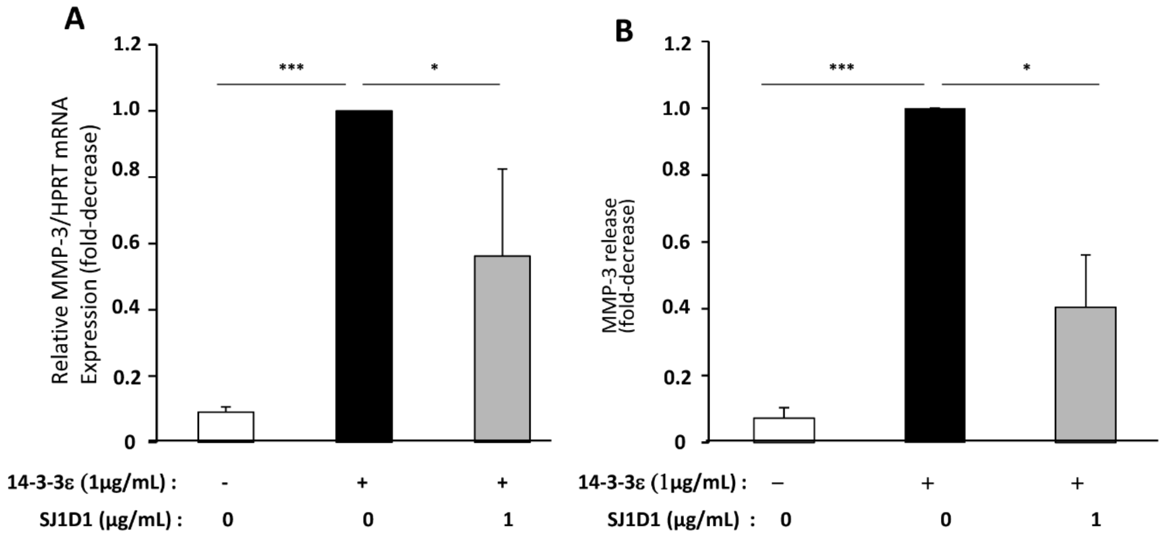

Figure S2: Inhibition of the expression and release of MMP-3 in human chondrocytes by CD13/APN blocking antibody. Primary cultures of human chondrocytes from patients with OA were starved, then stimulated with 1 µg/ml recombinant 14-3-3ε in the presence of a CD13 blocking antibody (SJ1D1) at 1 µg/ml for 24 h. MMP-3 expression was analyzed by RT-PCR **(A)** and ELISA **(B).** 14-3-3ε stimulated cells released 3580.9 ± 1456.2 ng/ml of MMP-3. Data are mean±SD relative decrease in MMP-3 release compared with stimulated cells (set to 1) from three independent experiments. *p<0.05; ***p<0.001.

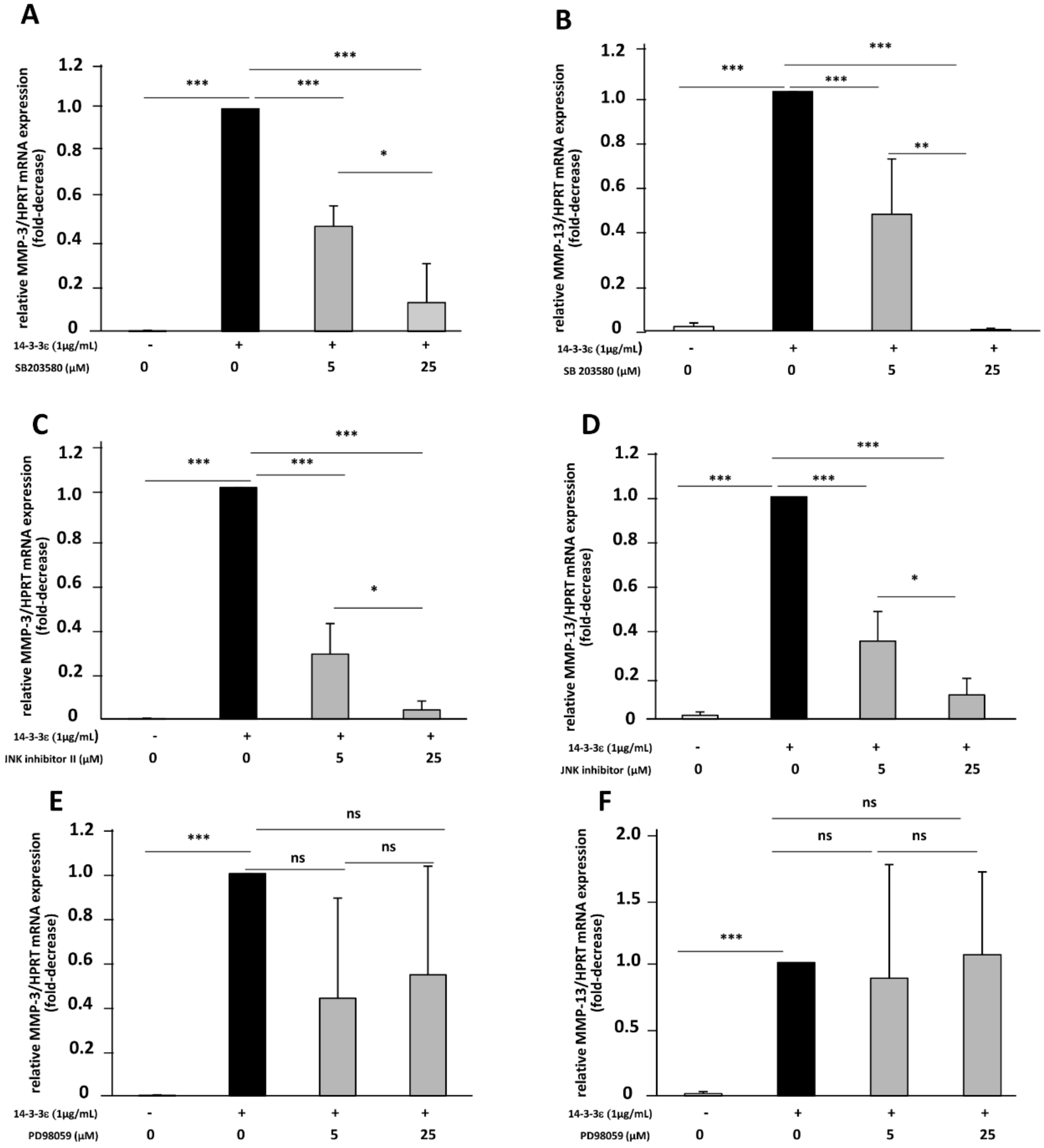

Figure S3: Involvement of MAPK in in 14-3-3ε-induced MMP-3 and MMP-13 expression by murine articular chondrocytes. Primary cultures of mouse chondrocytes were starved, then stimulated with 1 µg/mL recombinant 14-3-3ε for 24 h in the presence of specific inhibitors at 5 and 25 µM of MAPK (SB203580 for p38 MAPK, PD98059 for ERK1/2 and JNK inhibitor II for JNK kinase). MMP-3 **(A, C and E)** and MMP-13 **(B, D and F**) expression were analyzed by RT-PCR**.** Data are mean±SD relative decrease in MMP-3 and MMP-13 expression compared with stimulated cells (set to 1) from three independent experiments. ns: not significant; $^{*}p<0.05$; $^{**}p<0.01$; $^{***}p<0.001$.
